# Epidural Spinal Electrogram Provides Direct Spinal Recordings in Awake Human Participants

**DOI:** 10.3389/fnhum.2021.721076

**Published:** 2021-10-26

**Authors:** John F. Burke, Nikhita Kunwar, Maria S. Yaroshinsky, Kenneth H. Louie, Prasad Shirvalkar, Paul Su, Melanie Henry, George Pasvankas, Lawrence Poree, Lines Jacques, Doris D. Wang

**Affiliations:** ^1^Department of Neurological Surgery, University of California, San Francisco, San Francisco, CA, United States; ^2^Department of Anesthesia and Pain Management, University of California, San Francisco, San Francisco, CA, United States; ^3^Department of Neurology, University of California, San Francisco, San Francisco, CA, United States

**Keywords:** spinal electrical stimulation, functional mapping, neuromoduation, spine–physiology, spinal cord injury

## Abstract

Little is known about the electrophysiological activity of the spinal cord during voluntary movement control in humans. We present a novel method for recording electrophysiological activity from the human spinal cord using implanted epidural electrodes during naturalistic movements including overground walking. Spinal electrograms (SEGs) were recorded from epidural electrodes implanted as part of a test trial for patients with chronic pain undergoing evaluation for spinal cord stimulation. Externalized ends of the epidural leads were connected to an external amplifier to capture SEGs. Electromyographic and accelerometry data from the upper and lower extremities were collected using wireless sensors and synchronized to the SEG data. Patients were instructed to perform various arm and leg movements while SEG and kinematic data were collected. This study proves the safety and feasibility of performing epidural spinal recordings from human subjects performing movement tasks.

## Introduction

The spinal cord is more than a passive conduit of sensorimotor information between the musculature and the brain; it contains complex neural circuitry capable of integrating top-down cortical control of movement with bottom up sensory and biomechanical information from the periphery. An example of such an intrinsic spinal neural circuit is the lumbosacral “central pattern generator” (CPG) (Grillner and Wallen, 1985; MacKay-Lyons, 2002), which is a theoretical circuit in the caudal aspect of the spinal cord capable of autonomously generating stereotyped movements involved with locomotion (Dimitrijevic et al., 1998). Patterned epidural stimulation of the CPG in the lumbosacral spinal cord has been shown to restore the ability to walk in paralyzed patients suffering from spinal cord injury (SCI) (Angeli et al., 2018; Gill et al., 2018; Wagner et al., 2018). Although little is known how such stimulation interacts with endogenous spinal neural activity, a better understanding of the neurophysiology of intrinsic spinal neural circuits is essential for developing therapies for those suffering from motor or sensory dysfunctions of the spinal cord.

Currently, our understanding of intrinsic spinal circuits in humans is limited largely because there is no direct way to measure spinal electrophysiological activity in neurologically-intact patients performing motor tasks. To address this, we developed a novel method to study neurophysiological activity of the spinal cord during volitional motor and sensory tasks in humans by recording from externalized spinal epidural electrodes in patients undergoing spinal cord stimulation (SCS) evaluation to treat chronic pain. Here, we present our protocol for recording human spinal electrogram (SEG) (Shimoji et al., 1971) to advance our understanding of spinal neurophysiology.

## Materials and Methods

### Patient Selection

**FIGURE 1:**
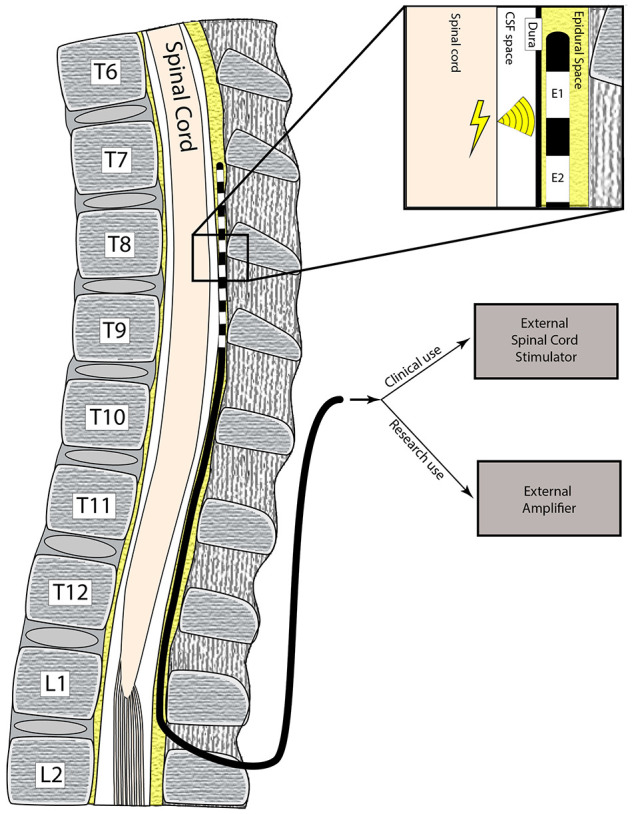
Schematic of electrode implantation. The patient has a surgical procedure to implant electrodes in the epidural space, which are used to record electrical impulses from the spinal cord. These electrodes exit the skin and are connected to the recording adapters and hardware to obtain SEG signals.

All adult patients with chronic pain undergoing a spinal cord stimulator (SCS) trial from the Pain Management Center and the Department of Neurological Surgery at the University of California, San Francisco were recruited for the study. For the SCS trial, epidural (distal) electrodes are placed on the spinal dura and the proximal end either exits the skin directly in the patient’s back (Figure 1), or it is connected to another lead extender which then exits at the skin. The trial period typically lasts 7–10 days during which a temporary stimulator is connected to the externalized leads to determine the efficacy of SCS.

Inclusion criteria included patients > 18 years, patients without previously existing neurological injury, and the ability to perform motor and sensory tasks and follow task instructions. Patients who had known motor deficits were excluded from the study. All study protocols were approved by the institutional review board (IRB) and all patients provided informed consent for participation (IRB# 9-29038).

### Electrode Selection

**FIGURE 2:**
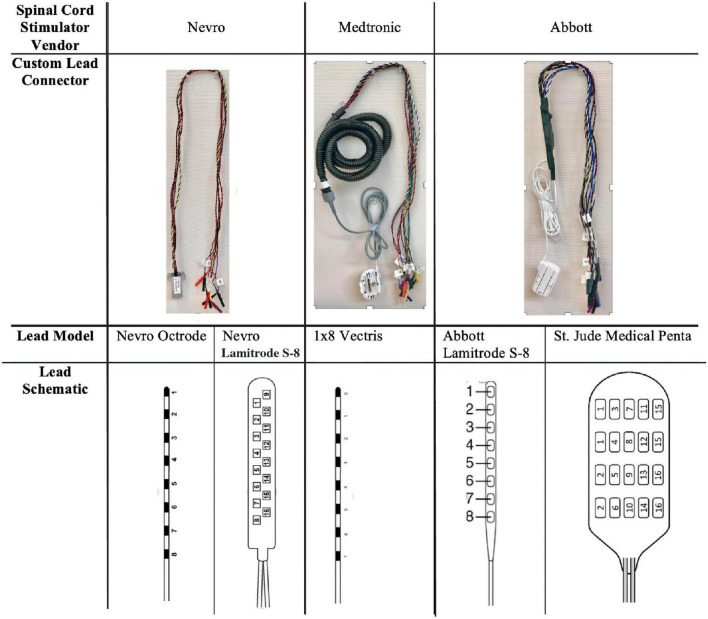
Lead types used in this study and customize connectors. The difference types of leads (based on medical device company) are shown. The top row demonstrates the customized connectors that were used to couple the leads to the external recording system. The bottom row demonstrates the schematics of the leads used in the study.

FDA-approved, commercially-available epidural electrodes for SCS, either in a “cylindrical” or “paddle” configuration, were used at the discretion of the subjects’ treating physician. Specifically, the following epidural electrode models were used: eight contact Octrode^*TM*^ lead (Nevro Corp., Redwood City, CA, United States), eight contact Lamitrode^*TM*^ S-8 lead (Abbott Neuromodulation, Austin, TX, United States), eight contact 1 × 8 Vectris^*TM*^ lead (Medtronic, Minneapolis, MN, United States), 16 contact Lamitrode 88 lead (Nevro Corp., Redwood City, CA, United States), and 16 contact Penta^*TM*^ lead (St. Jude Medical, Neuromodulation Division, Plano, TX, United States). Schematics of each lead type are shown in Figure 2 (bottom-panel). These electrodes were attached to custom-made connectors (Figure 2, top panel) which coupled the electrodes to the external amplifier during the research recordings. For the purposes of this paper, the entire implant is referred to as a “lead,” and each epidural contact on the lead is referred to as an “electrode.” Each lead contained either 8 or 16 electrodes.

### Operative Procedure

**FIGURE 3:**
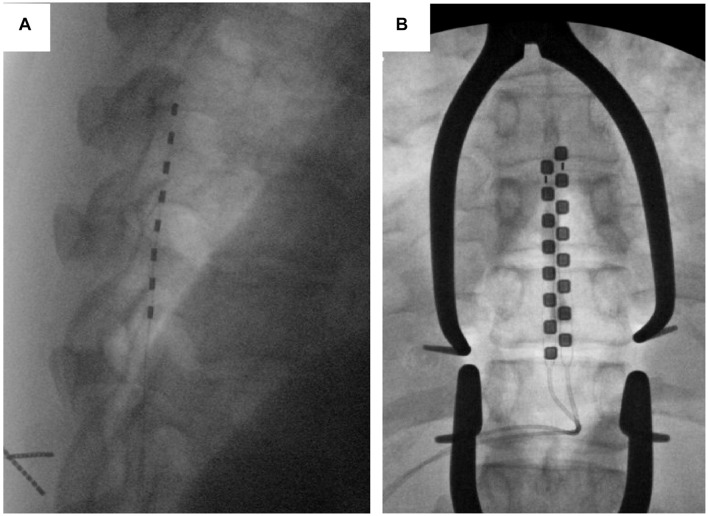
Lead types based on fluoroscopic imaging. An example of a cylindrical **(A)** and paddle **(B)** leads are shown using intra-operative fluoroscopic imaging.

Cylindrical leads were placed percutaneously under local anesthesia and monitored anesthesia care (MAC), where an introducer is placed in the epidural space and the lead is tunneled to the desired location. Paddle leads were placed under general anesthesia, where a midline incision was made and subperiosteal dissection performed to expose the lamina. A laminotomy was placed 1–2 spinal levels below the intended target and the paddle lead was inserted at the laminotomy and advanced cranially. For both lead types (cylindrical and paddle), fluoroscopic localization was used to position the leads at the spinal level of interest (Figure 3), which was selected in order to achieve optimal pain control. After placement in the epidural space, the proximal ends of the leads were tunneled through the skin.

### Research Recording Protocol Overview

After this trial period, the patients would then return to the hospital to have the leads removed, with the permanent device implanted at a later date. For patients with a planned lead removal (cylindrical electrodes), we conducted the study on the day of explant. For patients with paddle leads, we conducted the study either on the day of initial paddle implantation or the following day.

**FIGURE 4:**
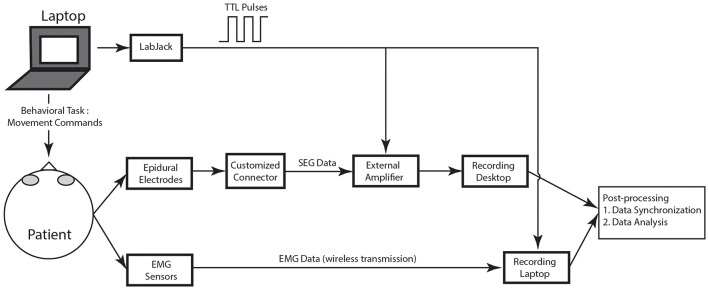
Schematic of recording protocol. In the schematic, the various modules of the recording protocol are shown.

Figure 4 shows an overview of the recording protocol, with box diagrams indicating the SEG and EMG source data, the sensors, and the connections needed to convert the recorded data to usable data format. Each of these modules are described in detail in the sections below.

### Spinal Electrograms Recordings and Customized Connector Cables

Spinal electrograms data were recorded by first disconnecting the external stimulator and connecting the lead to a customized “connector cable.” This custom connector cable was created using a proprietary external connector that was supplied by the electrode manufacturer (see Figure 2, top panel). In all cases, the connector was electrically coupled to retractable sheath banana protected “pin plugs.” Specifically, these extension cable connections were created using a small piece of perforated circuit board material with 16 individual wires connected to the protected pin plugs. Once all the wires were connected, the circuit board was covered with heat shrink tubing and an ohmmeter was used to trace each wire and applied the corresponding label to the pin plugs to verify the connection. Once the connector was in place, the protected plugs each separately contained passive connections to each electrode on the epidural lead. These pin plugs were then manually connected to the external amplifier of the recording device.

### External Amplifier

The pin plugs of the customized connectors are inserted into an external amplifier, which was either a 128 channel TDT (Tucker Davis Technologies, Inc., Alachua, FL, United States) PZ5 Neurodigitizer amplifier optically connected to a TDT RZ5D Bioamp processor (input impedance 10 kOhms) or a Neuro-Omega (Alpha-Omega Engineering, Nof HaGalil, Israel) clinical amplifier system. We used one of the electrodes as reference to reduce noise; per protocol, we used the cranial most electrode as the reference. For the Neuro Omega, the SEG signals were band-pass filtered between 1 and 1,024 Hz and sampled at 2,570 Hz. For the TDT, the SEG signals were band-pass filtered between 1 and 1,024 Hz and sampled at 3,051 Hz. Of note, the amplifiers were mains-powered, optically coupled to the recording devices, and contained no automatic artifact blanking. The amplifiers had no shielded enclosure. Of note, the PZ5 Neurodigitizer was run off battery power, however, the Neuro-Omega and the recording computers were connected to the main wall power during recording.

### Electromyography Recordings

Surface electromyography (EMG) data were recorded using eight external wireless sensors that were attached to muscle groups in the triceps, biceps, quadriceps, and hamstrings area using Trigno Avanti^*TM*^ portable EMG sensors (Delsys, Inc., Natick, MA, United States). These sensors are commercially available, have on-board power sources and Analog to Digital converters, and wirelessly stream digitized data measuring EMG activation and limb position to a dedicated recording laptop. The recording laptop was a separate PC station running EMGWorks^*TM*^ (Version 4.7.9, Delsys, Inc., Natick, MA, United States) that communicated with the Trigno sensors using a radiofrequency link/receiver system. These data were recorded during the task on this external laptop. All EMG signals were then subsequently downsampled to 256 Hz.

### Behavioral Task

**FIGURE 5:**
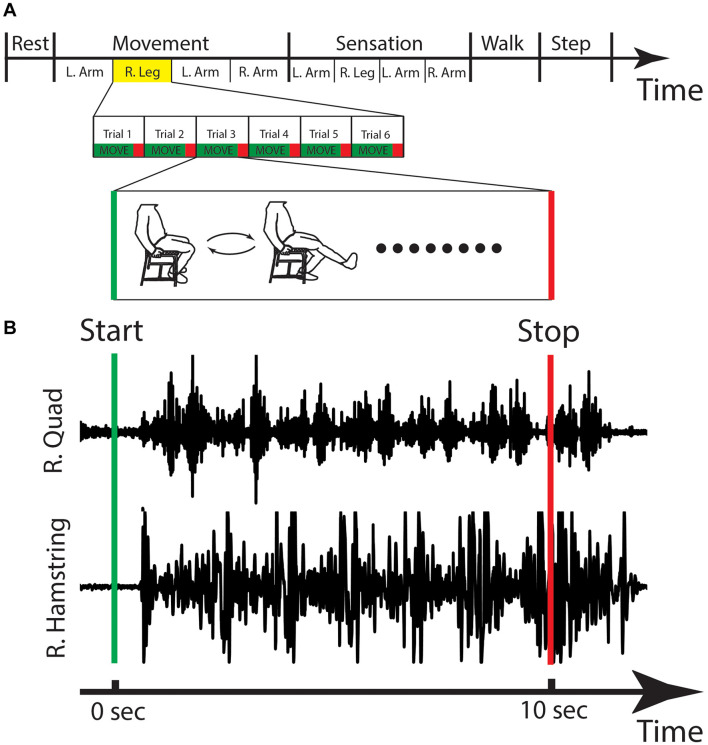
Behavioral task and EMG. **(A)** The stages of the behavioral task are shown, with the right leg movement highlighted and expanded in the lower panels with a cartoon figure of the type of movement that is done during this stage. **(B)** The EMG activity for one cycle of right leg movement showing the activations of the right quadricep (R. Quad) and the right hamstring (R. Hamstring). Sec, seconds; L, left; R, right.

During each recording session, the patient was shown a behavioral task that gave the patient visual commands to move. The behavioral task was written using custom Python^*TM*^ (Python Software Foundation, Wilmington, DE, United States) software using the PyEPL toolbox (Geller et al., 2007) that was implemented on a separate laptop. This laptop was placed in front of the patient at a distance of 36–48 inches and displayed instructions to move the limb. The task began with a 30 s rest period, during which the patient was instructed to sit still in a relaxed position. This rest period was followed by a period of controlled movement of each limb. The bilateral arms and legs were sequentially selected (in random order) for movement, and in each case the patient was asked to flex and extend either their elbow or knee continuously for 10 s. This was repeated six times (60 s of total movement for each limb) with a 5 s rest in between each period of motion. After six cycles of flexion/extension were completed for one limb, the patient was asked to perform the same sequence of events for each subsequent limb until all limbs were completed. A sensation task was them performed, where the researcher touched the patient’s limb (forearm or distal leg) for 10 s on each limb. Again, the order of limb stimulation was randomized. After the movement and sensation tasks, the patient walked around the room for 30 s (self-paced). Finally, the patient stepped in place for 30 s (self-paced) (Figure 5A). Figure 5B shows an example of EMG activity showing onset of right leg movement.

### Synchronization of Spinal Electrograms, Electromyography, and Behavioral Data

To synchronize multiple data streams, transistor-transistor logic (TTL) pulses were sent from a LabJack U3-LV^*TM*^ (LabJack Corporation, Lakewood, CO, United States) during the recording session, and the pulses were recorded by the external amplifier and the Delsys computer. The TTL pulses were created by custom code implementing the behavioral task, and pulse times were based on the CPU time of the laptop executing the behavioral task. We used a thresholding technique to find these same pulses captured by both the EMG and the external amplifier and used a regression technique to synchronize the TTL pulses times from the behavioral laptop with the sample times on both the amplifier and the EMG system. Custom code was used to align these TTL pulses off-line to synchronize the data.

### Data Analysis

Preprocessing: SEG and EMG data were downsampled to 256 Hz using offline signal processing tools that were custom written using Matlab^*TM*^ (Mathworks Inc., Natick, MA, United States). SEG data were passed through Butterworth notch filters centered around 60, 120, and 180 Hz to reduce 60 Hz electrical noise and its harmonics. SEG signals were re-referenced using bipolar montage between adjacent contacts to further reduce noise.

Power spectral density: For the power spectral density, we windowed the SEG data into 10 s intervals, which was chosen because it was the length of time the patient was asked to move for an individual muscle group. A 1-s buffer was included on either side of this window, and a bank of 30 Morlet wavelets (wave number 7) spaced from 2 to 40 Hz was used to calculate the instantaneous power at each time point. To calculate the power spectral density (PSD), these power values were averaged across time for each time window of interest. The power from 2.5 to 40 Hz was manually inspected to ensure there was not any EKG artifact as well as 60 Hz line noise.

To calculate the power spectra for the event-related comparison of upper extremity movement vs. rest, we similarly used a bank of 30 Morlet wavelets (wave number 7) spaced from 2 to 40 Hz and averaged these power values across time. In this case, smaller event related windows were chosen (2 s duration) surrounding each movement event, and similar sized, non-overlapping, windows were also chosen during rest activity. The power spectra were calculated during these windows, and averaged across all windows for the two conditions: movement vs. rest.

## Results

We recorded SEG from 10 patients (see Table 1); five patients were implanted with cylindrical electrodes and five patients with paddle electrodes. 56 electrodes were placed in the cervical and 44 placed in the thoracic spine. Of note, the recordings in 2/10 patients exhibited technical errors that were excluded from further analysis.

**TABLE 1:** Patient table.

**Subject ID**	**Spinal cord stimulator vendor**	**Lead type**	**Lead location**
1	Abbott	Lamitrode S-8	Cervical Thoracic
2	Abbott	Lamitrode S-8	Thoracic
3	Abbott	Lamitrode S-8	Cervical
4	Nevro	Octrode	Cervical
5	Medtronic	1 × 8 Vectris	Cervical
6	Nevro	Lamitrode S-8	Thoracic
7	Nevro	Lamitrode S-8	Cervical
8	Abbott	Penta	Thoracic
9	Nevro	Octrode	Cervical
10	Nevro	Lamitrode S-8	Thoracic

*The table includes patient de-identified identification (left column), the spinal stimulation vendor that was implanted, the lead type (cylindrical vs. paddle), and the amplifier used.*

**FIGURE 6:**
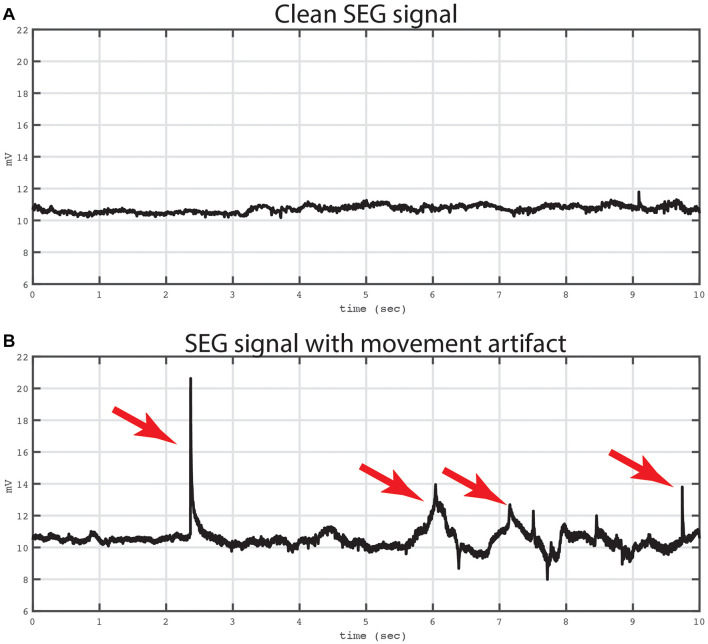
Example of bipolar recordings with and without artifact. Two traces from a bipolar recording are shown. Both traces were recording from a bipolar signal derived from electrodes in the cervical spine. The top trace **(A)** shows a 10 s period that is not contaminated by movement artifact. The Kurtosis value for this tracing was 2.29. The bottom trace **(B)** shows the same bipolar signal at later time window with mechanical artifact contaminating the signal. The artifact was evoked by mechanical manipulation of the leads and connector device relative to the patient. Artifacts are labeled with red arrows. The Kurtosis value for this tracing was 21.07.

We first verified that the data recorded from the spinal electrodes were of sufficient quality and free from noise. Figure 6 shows two example recordings of 10 s duration from a cylindrical lead in the cervical spine. Of note, both recording have been converted into a bipolar montage by subtracting the raw signal from one adjacent electrode. This method has been used extensively in intracranial invasive recordings to remove artifacts and common noise sources (Burke et al., 2013, 2014a,b). In this case, the bipolar montage was chosen to reduce potential movement and cardiac artifacts.

The top panel of Figure 6 shows an example of a recording that is not contaminated by movement or EKG artifact, and in the bottom panel of Figure 6 is an example from the same electrode showing an epoch contaminated by movement related artifacts, which was introduced by manipulating the lead and recording apparatus. To quantify the artifact on these two recordings, we used the Kurtosis measurement (Delorme et al., 2001) which measures the degree to which the data is peaked and sparse similar to movement related artifact. In addition, Kurtosis measurements have a standardized interpretation with values between 5 and 10 usually implying excessive noise contamination (Sederberg et al., 2007). In the current example, the Kurtosis of the top tracing was 2.29 and the bottom tracing was 21.07, respectively.

**FIGURE 7:**
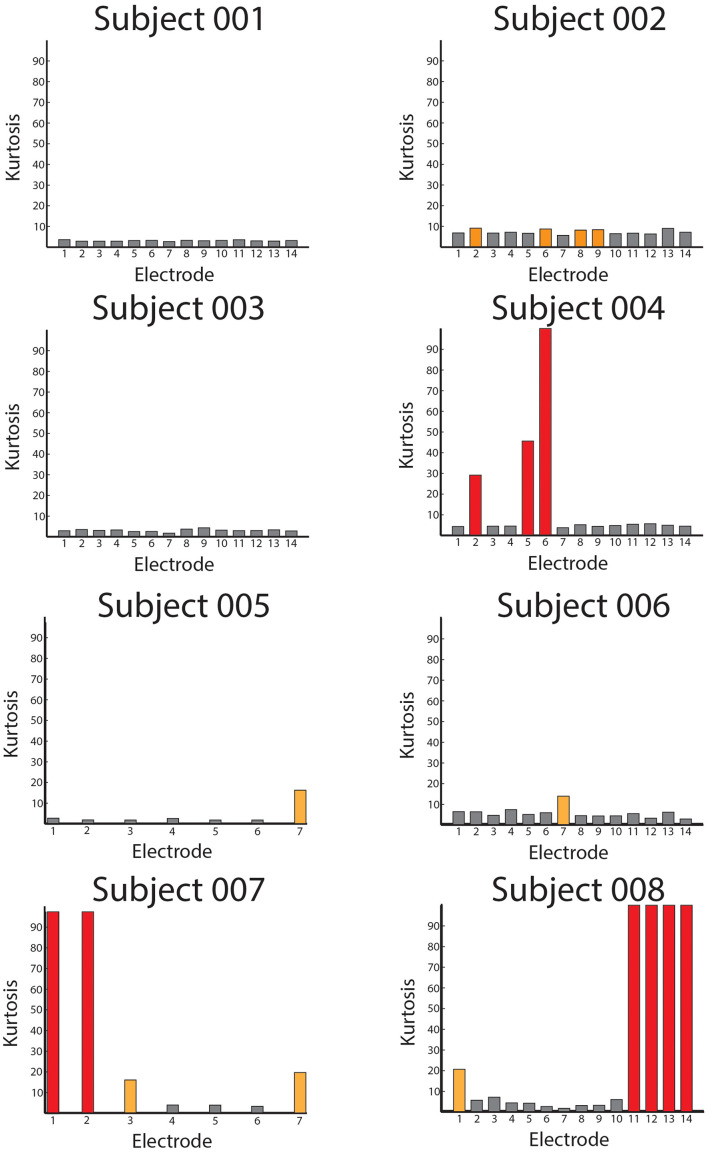
Averaged Kurtosis values across bipolar recordings. The average Kurtosis value for each bipolar recording is shown for every patient in the database. The red bars indicate Kurtosis values that were not usable as the recordings were saturated by mechanical artifact, and the orange values represent borderline Kurtosis values (>10) that were also not used in analyses.

Using the Kurtosis allowed us to summarize the data from all of the patients to quantify how free of overall noise the signals were. In Figure 7, we display Kurtosis values from all eight patients. The X-axis shows the electrodes from each patient, and the Y-axis shows the Kurtosis values for each bipolar pair. The Kurtosis was calculated on 10 s non-overlapping segments of data and averaged across these windows for the entire session to result in a single Kurtosis value for each bipolar pair. From the figure, it is clear that some of the bipolar pairs were heavily contaminated by noise with Kurtosis values greater than 100 (red bars). Other pairs were significantly contaminated by noise with Kurtosis values greater than 10 (yellow bars). However, the majority of the bipolar pairs had Kurtosis values < 10 and were considered useable data.

**FIGURE 8:**
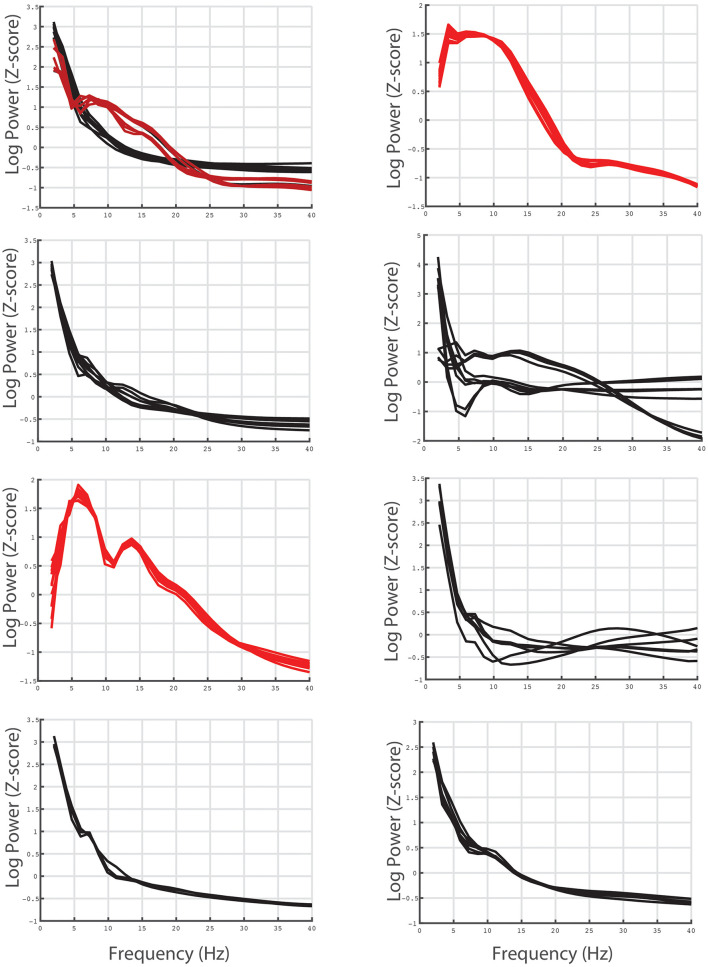
Averaged power spectra for each bipolar electrode pair recording for all individual subjects. The average power spectra for each bipolar recording (see text for calculation) are shown for all patients in the database. The red traces represent bipolar recordings derived from thoracic electrodes and the black traces represent bipolar recordings derived from cervical electrodes.

We next examined whether spinal recordings contained spectral information. Specifically, there have been no previous reports of neural oscillations in spinal circuits, or spectral decompositions of spinal electrophysiological data more generally. Thus, we wanted to establish whether SEG data (1) contained spectral activity similar to brain electrocorticography and (2) whether the features of these signals varied as a function of spinal location. Figure 8 shows the averaged power spectra for each bipolar recording. Similar to the Kurtosis calculation, we first segmented each recording into 10 s non-overlapping windows. We calculated the power spectra on these windows (see section “Materials and Methods”) and log transformed these power values and then whitened the distribution of power values across time windows (removed the mean and divided by the standard deviation). Finally, we averaged these log-transformed, normalized power values across time windows for each bipolar recording, which is shown in Figure 8. In the figure, each trace represents the power spectrum from one bipolar signal, averaged across all time. Of note, black traces represent bipolar recordings from cervical electrodes and red traces represent bipolar recordings from thoracic electrodes.

Figure 8 shows that SEG data shows overall similar spectral properties to brain invasive recordings, specifically there is a 1/f fall off of power relative to frequency. In addition, although some channels are contaminated by noise, there appears to be different shapes of the power spectrum for cervical compared to thoracic recordings. The consistency of the spectrum across anatomical location and individuals suggest that the current recordings reflect, at least in part, underlying spinal neural circuitry as opposed to noise or movement related artifact.

**FIGURE 9:**
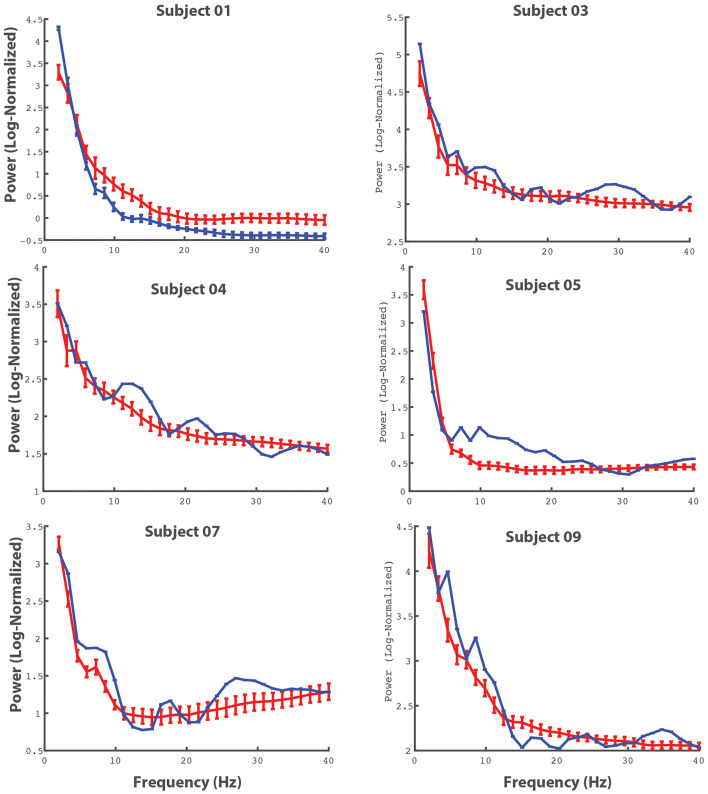
Averaged cervical power spectra during movement and baseline activity. The power spectra for individual electrodes (for all patients in with electrodes in the cervical spine) are shown. The red lines represent power spectra averaged across movement events (bilateral upper extremity elbow flexion) and the blue lines represent power spectra during periods of rest. The errorbars reflect 95% CI.

Given that our protocol was able to record SEG data with a sufficient signal-to-noise ratio, we next wanted to determine whether this SEG data contained movement related information. Although it is beyond the scope of this methodological paper to definitively show that spinal neural oscillations correlate with movement, we nonetheless wanted to provide preliminary data to demonstrate that recorded SEG data reflect behaviorally relevant phenomenon. To do this, we found the power spectrum for individual movements of the upper extremity (elbow flexion) using electrodes in the cervical spine and compared these power spectra to resting activity. Figure 9 shows these power spectra for selected electrodes for all patients with leads in the cervical region. From the figure, there is clear event-related spectral activity, which tends to show alterations in power in the theta and beta range during movement.

## Discussion

We demonstrate a novel method to record electrical potentials from the epidural space of neurologically intact humans. This method provides a feasible approach to obtain invasive spinal neurophysiological data from human participants during movement and sensation tasks (Crone, 1998; North et al., 2005; Lebedev and Nicolelis, 2006; Ramos-Murguialday et al., 2012).

SEGs could help define the spatial and temporal functional map of spinal motor neuron activation. Such spatially and temporally precise activation maps have been indirectly demonstrated when using patterned epidural spinal cord stimulation paradigms to restore gait functions after spinal cord injury (Wenger et al., 2016). However, the precise location of spinal motor units associated specific muscle activation has only been shown using retroviral tracers and anatomical studies of peripheral nerves to the spinal cord (Courtine and Sofroniew, 2019). By identifying the location and pattern of physiological activity from the spinal cord *in vivo* during normal movements, this protocol will aid in investigations that record direct spinal neurophysiology.

There are several limitations of the current study. First, it is difficult to separate sensory input from motor output in our task, and thus further work is needed to create tasks to differentiate motor from sensory SEG activity. Second, lead locations are chosen by the clinical team so as to best treat the patient’s chronic pain. Although this limits the type of information that can be collected, the resulting spinal invasive recordings represents useful data that can be used to investigate spinal cord function. Third, spinal recordings are clearly susceptible to movement artifact. As Figure 6 shows, several electrodes were not usable as the Kurtosis values were too high reflecting excessive artifact. The reasons why some electrodes are contaminated by noise is not entirely clear, however, it is likely that detecting spinal neural signals through the epidural space imparts greater propensity for motion of the CSF to influence the spinal signal. The degree to which this noise is prohibitive will depend on the application, however, it is very important for investigators to be cognizant of this limitation.

Despite these limitations, the opportunity to obtain direct spinal recordings from human patients without spinal pathology can advance our understanding of spinal cord physiology and circuit. Knowledge gained from SEG recordings will have direct applications in restoring functions to those suffering from neurological disease and injury.

## Conclusion

Our protocol demonstrates a feasible method to obtain SEG recordings from humans subjects during normal motor activity. SEG is a useful tool to study the physiological and functional organization of spinal cord.

## Data Availability Statement

The raw data supporting the conclusion of this article will be made available by the authors, without undue reservation.

## Ethics Statement

The studies involving human participants were reviewed and approved by UCSF IRB# 9-29038. The patients/participants provided their written informed consent to participate in this study.

## Author Contributions

DW provided the resources, performed the research, analyzed the data, and wrote the manuscript. JB performed the research, analyzed the data, and wrote the manuscript. NK, MY, and KL performed the research and wrote the manuscript. PSh, PSu, MH, LJ, LP, and GP provided the resources and wrote the manuscript. All authors contributed to the article and approved the submitted version.

## Conflict of Interest

The authors declare that the research was conducted in the absence of any commercial or financial relationships that could be construed as a potential conflict of interest.

## Publisher’s Note

All claims expressed in this article are solely those of the authors and do not necessarily represent those of their affiliated organizations, or those of the publisher, the editors and the reviewers. Any product that may be evaluated in this article, or claim that may be made by its manufacturer, is not guaranteed or endorsed by the publisher.
